# Safety and feasibility of an interactive workshop and facilitated outdoor walking group compared to a workshop alone in increasing outdoor walking activity among older adults: a pilot randomized controlled trial

**DOI:** 10.1186/s40814-018-0367-4

**Published:** 2018-11-29

**Authors:** Ruth Barclay, Sandra Webber, Jacquie Ripat, Theresa Grant, C. Allyson Jones, Lisa M. Lix, Nancy Mayo, Cornelia van Ineveld, Nancy M. Salbach

**Affiliations:** 1 Department of Physical Therapy, College of Rehabilitation Sciences, University of Manitoba, R106-771 McDermot Ave., Winnipeg, Manitoba R3E 0T6 Canada; 2 Elisabeth Bruyère Hospital, Ottawa, Ontario Canada; 3 Department of Physical Therapy, University of Alberta, Edmonton, Alberta Canada; 4 Department of Community Health Sciences, University of Manitoba, Winnipeg, Manitoba Canada; 5 Department of Clinical Epidemiology, McGill University, Montreal, Quebec Canada; 6 College of Medicine, University of Manitoba, Winnipeg, Manitoba Canada; 7 Department of Physical Therapy, University of Toronto, Toronto, Ontario Canada

**Keywords:** Outdoor walking, Walking group, Older adult, Workshop, Randomized controlled trial, Walking barriers, Accelerometry, Community

## Abstract

**Background:**

Limited outdoor walking is a marker of frailty and a risk factor for decline in mobility and self-care functioning, social isolation, and reduced health-related quality of life (HRQL). Objectives were to evaluate the safety, feasibility, and preliminary effect of a supervised outdoor walking group and interactive workshop compared to the workshop alone in increasing outdoor walking activity and identify an optimal method for estimating outdoor walking activity among older adults who infrequently walk outdoors.

**Methods:**

A pilot 2-parallel-group randomized controlled trial was conducted. Adults aged ≥ 65 years who reported walking ≤ 20 min/week outdoors were randomized in a 2:1 ratio to receive the GO-OUT program (1-day workshop and 9-week outdoor walking group), or the workshop alone. An external site conducted the randomization after workshop completion. The eight workshop activity stations aimed to build knowledge and skills to safely walk outdoors. The group-based outdoor walking program consisted of repetitive practice of mobility tasks at local parks. The primary outcome of outdoor walking activity used an activity monitor and GPS; secondary outcomes included aerobic, balance, and walking capacity; physical activity; participation; mood; and HRQL. Blinded outcome assessors evaluated participants at 0, 3, and 6 months. Qualitative interviews occurred after 3 months; data were analyzed with qualitative description. Quantitative data were summarized using descriptive statistics.

**Results:**

Forty-eight individuals were screened; 9 were eligible and randomized to the GO-OUT (*n* = 6) or workshop (*n* = 3) group. Data from 9 participants were analyzed. Mean age was 77 and 74 years in the GO-OUT and workshop groups, respectively. No falls occurred during the workshop and outdoor walking program. Average attendance of the walking group was 61%. All participants attended the evaluations and workshop. An analysis method combining data from activity monitors and GPS was developed to estimate outdoor walking. Themes from the qualitative analysis included the barriers to outdoor walking, impact of the workshop and GO-OUT walking group, and feasibility and acceptance of the assessment and intervention strategies.

**Conclusions:**

The trial protocol was deemed safe and feasible. Results were used to inform changes to the protocol to conduct a full-scale study.

**Trial registration:**

Clinical Trials.gov: NCT02339467.

## Background

Many older adults do not regularly walk outdoors. In fact in Canada, more than 42% of individuals aged 65 years and older walk outside fewer than 3 days per week [1]. Limited outdoor walking is a health concern because it is a marker of frailty [2] and a risk factor for decline in mobility and self-care functioning, social isolation, and reduced health-related quality of life (HRQL) [3, 4]. Furthermore, decreased walking activity predicts greater healthcare utilization in older adults [5]. With the growing aging population, interventions to increase outdoor walking frequency are essential to maintain health and independence.

To improve participation in outdoor walking, behavioral interventions should target modifiable barriers that prevent outdoor walking as experienced by older adults. *Personal or internal barriers* include difficulty walking which affects 33% of individuals aged 65–79 years and 55% of individuals over 80 years of age [6]. Fear of moving outdoors was reported by 29% of male and 65% of female community-dwelling older adults [2]. Limited leg strength, diminished balance, decreased balance self-efficacy, lower education, and inadequate access to a car can also limit outdoor walking [2, 7]. Many internal barriers are modifiable through regular practice walking outdoors where contextually relevant (task-oriented) opportunities and self-efficacy theory can be used to improve physical ability and self-efficacy to walk in the community [8, 9].

*External or environmental barriers* that limit outdoor walking among older adults include long walking distances to destination, time limits or attentional demands (e.g., walk signals, crowds), environments that require posture changes (e.g., stairs), poor neighborhood walkability (e.g., high levels of traffic, difficult terrain, safety), and inclement weather (e.g., temperature extremes, precipitation, snow, ice, wind, humidity, air quality) [7, 10].

Walking outside the home has health and participation benefits for older adults. For example, walking outdoors at least once a week has been associated with achieving more time spent in moderate-intensity physical activity than walking indoors [11]. Dual task practice, such as walking while talking, can build self-efficacy and capacity for community ambulation [12]. The capacity to walk outdoors also provides a means to participate in meaningful activities, such as shopping, and leisure activities (e.g., visiting friends, pleasure walking). Outdoor walking is associated with improved self-rated health and HRQL [11, 13, 14], and walking in outdoor natural environments improves mental well-being more than walking indoors [15].

Although a number of age-related changes contribute to walking difficulties [16–18], there is evidence that walking ability among older adults is modifiable through training interventions [19]. For example, an indoor trail walking program improved complex task walking and decreased fall risk compared to a regular walking program [20]. It has been argued that future interventions to improve walking abilities in older adults should consider a task-oriented motor learning approach [21]. Drawing from interdisciplinary fields of research, these authors describe the need to develop new compensatory strategies in order to maintain performance in context-relevant environments.

While numerous studies have aimed to increase physical activity among older adults via walking interventions [19], it is the premise of this study that interventions targeting internal barriers to outdoor walking have the potential for additional benefits. Preliminary work with stroke survivors indicates that outdoor walking interventions hold promise for improving community mobility skills [22, 23]. However, no studies have yet examined whether a walking program designed to target outdoor walking abilities is effective in improving community ambulation skills among older adults. Based on the evidence to date, this study posits that internal barriers are modifiable through regular practice of walking outdoors where contextually relevant opportunities to improve physical ability and self-efficacy to walk in the community are provided.

The purpose of this study was to evaluate the feasibility of a two-group randomized controlled trial (RCT) protocol designed to evaluate the effectiveness of an interactive workshop and outdoor walking group compared to the workshop alone in increasing outdoor walking activity (primary outcome) and physical capacity, walking self-efficacy, mood, physical activity, participation, and HRQL (secondary outcomes) among older adults who walk outdoors infrequently.

The objectives were to:

1. Identify the optimal analysis method of outdoor walking activity, using accelerometry and GPS (global positioning system)
2. Evaluate the preliminary effect and safety of a supervised outdoor walking group program and workshop compared to the workshop alone among older adults who report infrequently walking outdoors
3. Identify the feasibility and acceptability of the study protocol (recruitment, attendance, equipment, frequency of evaluation, acceptability of the outcome measurement scales and intervention)

## Methods

### Study design

A two-group mixed methods pilot RCT was conducted at a single site in Winnipeg, Canada [24]. Older adults who reported infrequently walking outdoors were randomly assigned, using a 2:1 ratio, to receive an interactive workshop and outdoor walking program, or the workshop alone. Primary and secondary study outcomes were evaluated at baseline (i.e., month 0, April 2015), 3 months (immediately post-intervention, July 2015), and 6 months (October 2015). Study participants in each group were invited to participate in a focus group or interviews within their intervention group at 6 months. CONSORT guidelines for reporting were followed.

### Conceptual framework

**Fig. 1:**
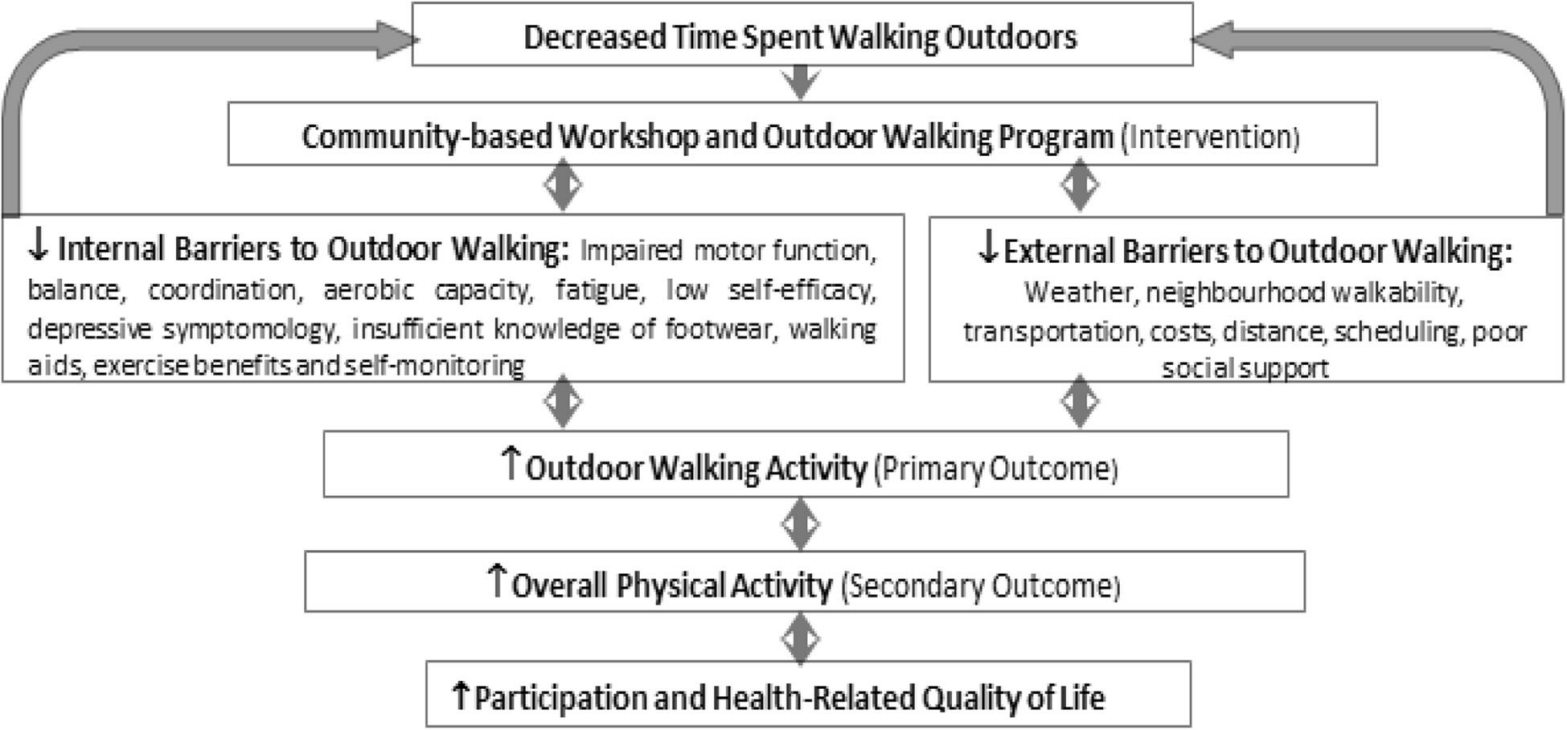
Conceptual framework for the GO-OUT intervention

The conceptual framework for this project (Fig. 1) was developed by NS and RB, based on the literature, associations between outdoor walking, physical activity, Bandura’s self-efficacy theory, the task-oriented approach, and other variables [8–10, 14, 25]. The workshop and walking group were designed to decrease internal and external barriers as a mechanism for increasing outdoor walking activity and time spent in physical activity. Increasing outdoor walking is expected to enable participation in meaningful activities and improve HRQL.

### Participants

Inclusion criteria were as follows: older adults aged ≥ 65 years, self-reported ability to walk continuously on a flat surface ≥ 1 block independently with or without a walking aid and without supervision, self-reportedly accumulating ≤ 20 min of outdoor walking in a typical week, mental competency, indicated by a score of ≥ 18 on the telephone version of the Mini-mental State Exam [26]. Exclusion criteria were as follows: active walkers accumulating ≥ 100 min of total (indoor and outdoor) walking per week; ≥ 2 falls in the prior 12 months or presented with an acute fall [27]; diagnosed with cardiac, respiratory, peripheral vascular, or other health conditions that would prevent safe and full participation in the interventions; receiving rehabilitation treatment such as physical or occupational therapy for goals related to walking; postural hypotension determined by measuring lying and standing blood pressure; severe limitations to visual acuity identified using a fall prevention screen; and resting heart rate under 45 or over 100 beats per minute.

To limit fall risk and ensure safety of participants, individuals were screened on the phone and then invited to a physical assessment, which included evaluation of postural hypotension, visual acuity, and resting heart rate [27]. The PAR-Q+ [28] (an evaluation of physical activity readiness) was completed by each participant. Each participant’s family physician was asked to review PAR-Q+ results and confirm that the participant was able to participate in physical activity.

### Recruitment

Potential participants were recruited using community-based recruitment strategies in March and April, 2015. We advertised through a seniors group that supports active living, the local newspaper, seniors’ centers, an organization supporting those recovering from stroke, and posters in seniors’ apartments. Phone and physical screening were completed and informed consent was obtained. If the physical screen was passed, a person continued with the baseline assessment.

### Measurement

Evaluators were trained in the use of the outcome measures; they were also unaware of group assignment. Evaluations of primary and secondary outcomes were completed in a university research lab, situated in a rehabilitation hospital.

The primary study outcome of outdoor walking activity was estimated by synchronizing data from an accelerometer (ActiGraph GT3X+ activity monitor (ActiGraph, Pensacola, FL)) and a global positioning system (GPS) monitor (Qstarz BT-Q1000XT A-GPS Travel Recorder). The GT3X+ includes a triaxial accelerometer to detect steps and activity counts. This monitor has been shown to be reliable [29] and valid for measuring physical activity under both laboratory [30] and free-living conditions [31]. The Qstarz A-GPS Travel Recorder was chosen for ease of use and capability for accurately recording GPS data (within 3 m) for up to 40 days [32]. The A-GPS Travel Recorder was configured using QTravel software (Qstarz, Taipei, Taiwan) to log GPS data every 5 s. Participants were asked to wear both monitors over the right hip on a belt around the waist during waking hours for eight consecutive days. Participants completed a log sheet noting when they donned and doffed the monitors each day.

Components of physical capacity that were expected to facilitate outdoor walking and total physical activity were evaluated. These components included aerobic capacity, balance, lower limb strength, and comfortable walking speed, and they were evaluated separately using the 6-min walk test (6MWT) [33, 34], the Berg balance scale [35], the 30-s sit to stand test [36], and the 10-m walk test [33], respectively. The Ambulatory Self-Confidence Questionnaire (ASCQ) [37] was used to evaluate walking self-efficacy. Mood was evaluated with the Geriatric Depression Scale short form [38, 39]. If a participant scored > 9, indicating depression, he/she was not excluded, but the family physician was notified. Physical activity, walking activity, and participation were evaluated using the Community Health Activities Model Program for Seniors (CHAMPS), a self-reported 40-item measure of social, leisure, and physical activity undertaken by older adults [40]. Specific questions are combined to produce subscales for time spent in MVPA, walking and total participation time per week. Health-related quality of life was assessed with the Rand-36 [41], a widely used generic measure of HRQL with eight subscales of physical, mental, and social functioning.

At each evaluation, participants were asked to identify additional interventions they were receiving. There was a risk of over-estimation in self-reporting the time spent in walking outdoors and total physical activity. This was mitigated by the use of a self-report questionnaire in addition to the use of the GPS and activity monitor that objectively measured walking activity. The use of both self-report and objective measures also strengthens the interpretation of results, allowing comparison of different data collection methods.

Data on age, sex, education level, social support, comorbidity, self-reported reasons for outdoor walking limitation, and neighborhood walkability were collected at baseline. Neighborhood walkability was evaluated using the 67-item self-report Neighbourhood Environment Walkability Scale (NEWS-CFA) [42, 43].

Intervention fidelity was captured by documenting attendance and completion of the workshop and walking group activities. The occurrence of adverse events during the workshop and outdoor walking sessions was monitored and documented using an adverse event form. Occurrence of injurious falls, defined as a fall resulting in injury requiring medical care, was tracked. Participants were provided with monthly falls logs and asked to record occurrence of falls over the course of the study; they were also called monthly to determine if any falls had occurred. If a fall occurred, the participant was questioned regarding the cause and circumstances of the fall and if there was an injury requiring medical care.

### Randomization

After the baseline assessment and interactive workshop, a research assistant in another city (ensuring allocation concealment) randomized participants using a 2:1 ratio by flipping a coin to the interactive workshop and outdoor walking program (termed the GO-OUT program) or the interactive workshop alone, respectively. The 2:1 ratio was chosen to enable us to gather increased information about the feasibility of the GO-OUT intervention. Although we planned to stratify as slow (gait speed < 0.8 m/s) or fast (gait speed ≥ 0.8 m/s) walkers prior to randomization, none of the participants walked slower than 0.8 m/s. One married couple that desired to be in the same group was enrolled in the trial. This couple was randomized as a dyad. ID numbers of the remaining participants were then drawn, and group assignment alternated until the workshop-only group had three; those remaining were placed in the GO-OUT program.

### Interventions

#### Interactive workshop

The 5-h interactive workshop incorporated a series of eight activity stations at which participants worked in small groups of two or three and learned information, strategies, and practiced skills to safely walk outdoors. Each station involved didactic teaching by a facilitator and activities to increase knowledge and skills related to the following: (1) the Canadian Physical Activity Guidelines for older adults [44]: the guidelines were discussed and participants determined the number of minutes of physical activity they completed in the previous week. (2) Setting SMART goals [45]: participants practiced writing outdoor walking-related goals that were specific, measureable, achievable, realistic, and timely. (3) Use of a pedometer [46–48]: participants were given a pedometer, taught how to use it, and practiced using it. (4) Use of Nordic walking poles [49, 50]: participants were instructed in and practiced adjusting poles and walking with the poles. (5) Appropriate footwear selection [51], foot care, and proper walking pattern and use of walking aids [8, 45]: participants watched a video of a typical gait pattern, watched a video of their own gait, discussed foot care, and looked at examples of good and poor walking shoes. (6) Fall prevention [52]: participants answered questions on a fall risk questionnaire and discussed ways to prevent falls at home and in the community. (7) Self-monitoring of exercise intensity [53]: the facilitator discussed safety when exercising, the signs of when to stop exercise, and how to gauge one’s own intensity of exercise. (8) Postural awareness and balance exercises: participants practiced a number of balance exercises while the facilitator gave each person individualized suggestions for making each exercise more or less challenging, as required.

The facilitators at each station were physiotherapy faculty and graduate or undergraduate students. All facilitators underwent training for the workshop stations at a training session, utilizing a prepared facilitator training guide which was developed to mirror the participant workbook. Each participant received a workbook which summarized information from each workshop station. The workbook was developed by the research team, using current research evidence, physical activity guidelines, clinical and research expertise of the team, and older adult preferences [54]. Participants were asked to use the workbook as an information resource and to record their outdoor walking goals. The pedometer was used by each participant for personal use and goal setting. All participants were encouraged to walk outdoors in their own neighborhoods with a partner, such as a family member or friend, for safety. Participants in the control group received the interactive workshop alone.

#### Outdoor walking program

Participants assigned to the experimental group received the interactive workshop followed by a 9-week facilitated outdoor walking program in summer months (i.e., the GO-OUT program). The walking program was group-based. Participants met twice a week for a 60-min session designed according to physical activity recommendations for older adults [44, 53]. Participants met at four city park locations that met the following selection criteria: public access, availability of benches to rest, and public washrooms. There were a variety of surfaces and environmental factors introduced to challenge the participants, e.g., carrying objects, diverting the walker’s attention, walking up and down curbs, slopes, and level or uneven surfaces [22, 55]. Each session included a 10-min warm-up, a planned walk in the park with a gradual increase of challenges, and a 10-min cool down [53]. Based on the needs of each group member, the physiotherapist facilitator gradually increased continuous walking exercise from a start of 10 min [53] to a maximum of 40 min, with increased difficulty [22, 56, 57]. Guidelines for each session were developed for gait speeds of ≥ 0.8 m/s and < 0.8 m/s. Balance and functional strengthening exercises were included as a component of the warm-up and planned walk, in recognition that multifactorial interventions are important in addition to walking for those who are at risk for falls [58]. Activities for each session were based on the Patla and Shumway-Cook dimensions of community mobility [10], with a focus on different dimensions each week. Dimensions include the following: distance, postural transitions (such as sit to stand), varied terrain, temporal factors (such as crossing a street in time), physical load, attentional demands, and traffic density (such as walking in crowds). Each dimension was targeted with activities in 2 or 3 weeks of the 9-week intervention. The same activity was repeated in the same week to reinforce the skills needed.

Participants were supervised based on a 1:3 facilitator-to-participant ratio to allow for assistance and individualization of the intervention where necessary. The lead facilitator was a physiotherapist with expertise in safe exercise training among individuals with chronic conditions. She was able to encourage participation, was trained in CPR, and carried a mobile telephone. The assistant was also a physiotherapist. The group setting provided peer and social support for the participants during the activities.

Pre-determined reasons to cancel the walking group for the safety of the participants included rain, thunder, or lightning; humidex (what the temperature feels like, taking humidity into account) above 30 °C [59]; temperature above 30 °C; temperature below 5 °C; wind speed or gusts above 30 km/h [59, 60]; and an insufficient ratio of instructors-to-participants to ensure safety, i.e., less than 1 instructor to 3 participants. Environment Canada recommends that outdoor activity be reduced or modified when the humidex is in the mid to high 30s, depending on the age and health of the individual, physical shape, clothing, and other weather conditions [59]. It has been shown that non-uniform winds should be less than 9 m/s (32.4 km/h) to avoid momentary loss of balance, but that performance is unaffected when wind gusts are below this speed [60].

### Focus groups and interviews

After the 3-month evaluation, all participants were invited to participate in face-to-face focus group or interviews conducted separately for each intervention group. The interview guide was based on the conceptual framework [25] developed by NS and RB (Fig. 1) and objectives 2 and 3 of this study. A qualitative descriptive approach with directed content analysis was used [61–63]. We aimed to explore participants’ perceptions of the study protocol, including the benefits, challenges, and potential strategies to improve recruitment; adherence to the interventions; intervention strategies and their components; and data collection procedures. Qualitative description was viewed as an appropriate means to gathering and analyzing the data of the study, as the intent was to seek an understanding of patterns or responses rather than subjecting the data to a high degree of interpretation. Interviews were facilitated by JR and RB, and were digitally recorded, and transcribed verbatim.

## Analysis

### 1. Identify the optimal method for estimating outdoor walking activity, using accelerometry/GPS

ActiLife6 software (ActiGraph, Pensacola, FL) was used to initialize the GT3X+ devices (100 Hz) and download and analyze the data (default filter). Participants with ≥ 4 valid days with a 10-h per day minimum time wearing the devices (wear time) were included in the analysis [64]. One-second epoch GT3X+ files were re-integrated into 5-s and 60-s files to allow for synchronization with the GPS data and analyses of activity bouts respectively.

Freedson cut-points were employed to define MVPA (≥ 1952 counts per minute (cpm)) using the 60-s epoch files [65]. MVPA was also measured using a Lifestyle cut-point (≥ 760 cpm) which captures activities ≥ 3.6 METs [66] and has been suggested to reflect older adults’ free-living moderate-intensity activity (for example, walking) [67]. Total time spent in MVPA (Freedson and Lifestyle cut-points) was determined along with identification of bouts of MVPA (≥ 10 min at or above Freedson MVPA threshold, ≥ 10 min and ≥ 5 min at or above Lifestyle cut-point). Lifestyle bouts were time matched with GPS locations to determine if the activity bout was indeed outdoor walking. The following parameters were determined from the Lifestyle bouts and GPS (there were only small numbers of Freedson bouts): location of bouts, total number of bouts, duration of each bout, steps per bout, and total walking time in bouts per day. Weekly bouts and minutes of MVPA were calculated by multiplying average bouts per day by 7. Steps per day were also calculated.

GPS data in Qstarz files were combined with the GT3X+ 5-s epoch files using ActiLife6 software to create time-synchronized CSV files (date, time, activity count, latitude, longitude, elevation, and speed data). To determine the location of activity bouts, the start and end time for each Lifestyle bout was noted and matched in the combined GPS/GT3X+ CSV file. If GPS data were available for the time period, then the GPS KML file was opened in Google Earth (Google, Inc., Mountain View, CA) and the latitude and longitude coordinates associated with the time period of the activity were used to identify the location of the bout and determine whether it occurred indoors or outdoors. For bouts that occurred outdoors, mean speed (km/h) was also calculated. Bouts were categorized as occurring indoors, outdoors or at an uncertain location (when the GPS location could not be determined).

### 2. Evaluate the preliminary effect and safety

As this was a pilot study, we did not test hypotheses related to the effectiveness of the GO-OUT intervention compared to the interactive workshop alone. Primary outcomes were presented as individual scores by group for each participant, assisting with determining feasibility of the analysis methods of the activity monitor and GPS analysis. For secondary outcomes, we used raw scores to calculate subscale and total scores; descriptive statistics summarized scores by group with medians and 25th and 75th percentile.

Safety was evaluated by determining the number, nature, and timing of adverse events, including injurious and non-injurious falls, occurring throughout the study.

### 3. Evaluate the feasibility and acceptability of the study protocol

#### Quantitative analysis

Feasibility of the study protocol was evaluated using the rate of interactive workshop and walking group attendance and completion of planned workshop and walking group activities. The percentage of interactive workshop stations finished, and average number of walking groups attended were calculated.

The number of individuals recruited per month and the withdrawal rate at each follow-up evaluation were calculated. The completion and ease of use of study measures and equipment (activity monitor, GPS) were also documented.

#### Qualitative analysis

For the qualitative analysis, a directed content analysis approach, using the study’s conceptual framework as an initial guide to analysis was used [63]. The transcripts were reviewed and coded individually by RB, NS, and JR. All three authors met jointly to discuss coding. Similar codes were grouped into categories based on the conceptual framework, while keeping open to the possibility of new categories that arose.

Rigor was addressed using triangulation: three researchers completed the data analysis, multiple interviews were undertaken and findings were reported with quotations of participants to support each category [68, 69].

#### Identifying challenges in the pilot

Using both quantitative and qualitative results, the first two authors discussed challenges that occurred and determined changes to the protocol for a full study.

## Results

**Fig. 2:**
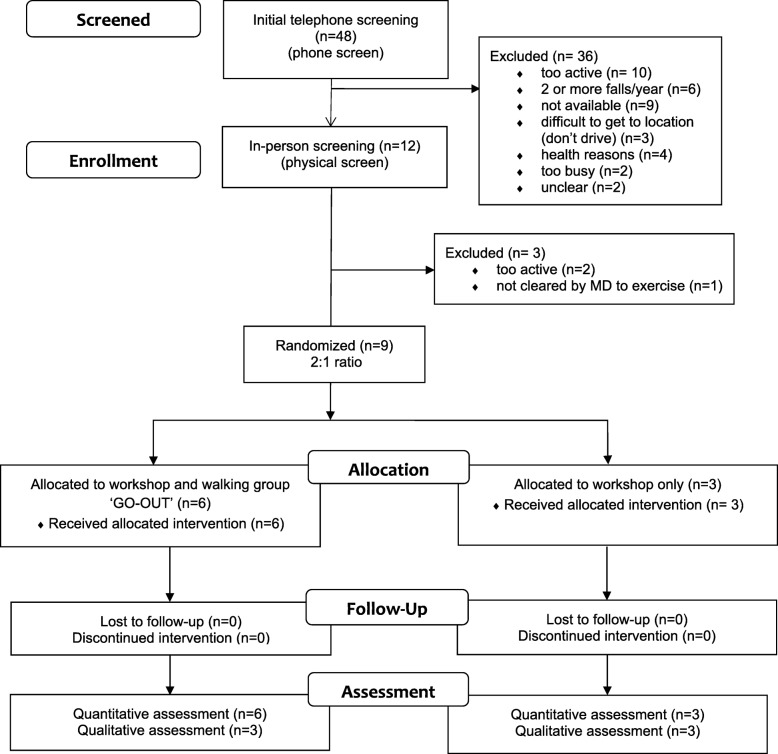
CONSORT diagram—extension for pilot and feasibility trials

Forty-eight individuals were screened for eligibility. Nine participants were included and randomized. (Please see Fig. 2 for the CONSORT diagram.) Six participants were randomly assigned to the GO-OUT intervention, and three to the interactive workshop alone. All participants remained in the group to which they were assigned. The demographic characteristics of the participants in each group are described in Table 1. The mean (standard deviation) age of participants was 77 (3) years in GO-OUT and 74 (8) years in the interactive workshop-only group. All participants were retired from paid employment, and only one participant in the walking group used a walking aid (a four-wheeled walker). All participants drove, except for one woman in the walking group, who was able to utilize local accessible public transport to all of the GO-OUT intervention locations.

**Table 1:** Demographic characteristics of participants at baseline

Variable	GO-OUT group (*n* = 6)		Workshop group (*n* = 3)	
Female *n* (%)	4	67	3	100
Self-rated health *n* (%)				
Excellent	1	17	0	0
Very good	0	0	1	33
Good	4	67	2	67
Fair	1	17	0	0
Poor	0	0	0	0
Chronic conditions *n* (%)				
Hypertension	4	67	1	33
Heart attack	1	17	0	0
Asthma	2	33	0	0
Arthritis	4	67	1	33
Ulcer disease	1	17	1	33
Diabetes	1	17	0	0
Glaucoma	1	17	2	67
Impaired hearing	4	67	1	33
Other	1	17	2	67
Age (years), mean (SD)	77	3	74	8
Number of medications mean (SD)	5	4	6	3
Minutes walked outdoors per week (self-report from screening) mean (SD)	18	15	20	17
Times/week walking outdoors (self-report from screening) mean (SD)	1	1	1	1

### Identify the optimal analysis method of outdoor walking activity, using accelerometry and GPS

One participant in the GO-OUT group did not meet minimum activity monitor wear-time criteria at 3 and 6 months, so no valid activity monitor data were collected at these time points for this individual.

Because the number of Freedson activity bouts per day was zero and the number of Lifestyle bouts ≥ 10 min in duration was also < 1/day, we determined the weekly number of ≥ 5 min Lifestyle bouts as well as the total weekly sporadic Lifestyle MVPA minutes. The mean number of steps/day for all participants was 3893 (SD 1929) at baseline, and this value did not change at 3 months or 6 months. However, steps per day reflect all walking, including walking in the home, not specifically outdoor walking. For the outdoor walking outcome, data are presented at an individual level, by group (see Table 2). The Lifestyle bouts were categorized as occurring in indoor, outdoor, or unknown locations according to the GPS data.

**Table 2:** Primary outcome—outdoor walking activity measured by accelerometry and GPS

Outdoor walking activity	GO OUT						Workshop-only		
Participant	1	2	3	4	5	6	7	8	9
Days of wear time/8									
0 month	8	5	8	8	8	7	7	8	8
3 month	5	0	5	7	7	5	7	6	7
6 month	5	0	7	7	6	5	6	7	7
Lifestyle 5-min outdoor bouts (1 week)									
0 month	0.9	0	0	25.4	8.8	0	0	12.3	0.9
3 month	0	*	0	18	0	5.6	3	3.5	0
6 month	0	*	0	13	2.3	4.2	1.2	0	2
Lifestyle 5-min indoor bouts (1 week)									
0 month	5.3	7	0	5.3	0	12	0	43.8	3.5
3 month	19.6	*	0	10	1	4.2	0	89.8	4
6 month	8.4	*	2	8	1.2	4.2	0	22	4
Lifestyle 5-min unknown location bouts (1 week*)*									
0 month	0	0	0	0.9	0	7	2	0.9	0
3 month	0	*	0	2	1	2.8	3	0	0
6 month	0	*	0	2	0	5.6	0	5	0
Sporadic min of Lifestyle MVPA (1 week)									
0 month	43.8	229.6	59.5	430.5	245	466	143	878.5	161.9
3 month	296.8	*	51.8	485	153	252	171	1200.5	192
6 month	186.2	*	102	397	128.3	266	120.2	506	180
Steps/day									
0 month	4051	3268	1401	5154	5113	9465	2614	7338	2203
3 month	3802	*	1508	5701	4334	2442	9732	6803	2622
6 month	3911	*	1935	4432	3952	2511	2019	4712	2703

All participants had 0 Freedson and Lifestyle 10 min bouts

*MVPA* moderate to vigorous physical activity

*Insufficient wear time

### Evaluate the preliminary effect and safety

Median scores on measures of secondary outcomes are presented by intervention group in Table 3. No falls occurred during the interactive workshop or the outdoor walk sessions. Three participants in the walking group had one fall each while performing usual activities during the 6-month duration of the study; two saw a doctor after their fall. One of these participants experienced a fall while walking outdoors during a holiday that required a doctor’s visit. One person in the control group had one fall, and sought treatment from a chiropractor. No fractures, hospital visits, or long-term issues were reported as a result of the falls.

**Table 3:** Secondary outcomes

Measure (units, scoring)	GO-OUT group (*n* = 6) median P_25_, P_75_			Workshop group (*n* = 3) median P_25_		
	0 month	3 month	6 month	Baseline	3 month	6 month
6MWT (meters)	372.0 328.9, 480.9	381.0 340.0, 567.5	386.0 349.5, 477.0	426.5 340.5	367.0 330.0	390.0 255.0
Ambulatory Self Confidence Questionnaire (0–10)	7.8 7.2, 8.7	9.0 7.7, 9.9	8.8 6.9, 10.0	9.7 8.6	9.5 7.3	9.7 7.8
10mWT (meters/second)	1.1 1.0, 1.3	1.0 0.9, 1.2	0.9 0.6, 1.0	1.3 1.0	1.1 1.0	0.9 0.8
Berg Balance Scale (0–56)	53.5 50.0, 55.3	53.5 47.5, 56.0	51.0 45.5, 55.3	54.0 44.0	53.0 51.0	52.0 48.0
Geriatric Depression Scale (0–15)	0 0, 4.0	1.0 0,3.0	1.0 0,2.5	3.0 1.0	3.0 1.0	4.0 0
RAND-36 Physical Function (0–100)	52.2 31.3, 81.3	70.0 27.5, 81.3	65.0 40.0, 87.5	70.0 40.0	65.0 55.0	80.0 35.0
RAND-36 Role Physical (0–100)	50.0 0, 100.0	75.0 0, 100.0	25.0 0, 81.3	75.0 50.0	100.0 0	0 0
RAND-36 Role Emotional (0–100)	66.7 0, 100.0	100.0 91.7, 100.0	100.0 25.0, 100.0	66.7 0	0 0	33.3 0
RAND-36 Energy (0–100)	60.0 21.3, 81.3	60.0 38.8, 76.3	52.5 33.8, 76.3	55.0 15.0	70.0 25.0	55.0 30.0
RAND-36 Emotional Well Being (0–100)	72.0 54.0, 89.0	78.0 72.0, 83.0	80.0 70.0, 97.0	80.0 80.0	84.0 64.0	72.0 38.0
RAND-36 Social Functioning (0–100)	81.3 28.1, 100.0	81.3 50.0, 100.0	75.0 50.0, 100.0	75.0 50.0	75.0 37.5	75.0 37.5
RAND-36 Pain (0–100)	51.3 16.8, 75.0	58.8 16.9, 80.6	56.3 30.0, 65.0	67.5 47.5	57.5 57.5	57.5 45.0
RAND-36 General Health (0–100)	65.0 40.0, 71.2	47.5 38.8, 75.0	55.0 50.0, 65.0	55.0 50.0	65.0 55.0	65.0 40.0
Sit-to-stand (# in 30 s)	9.5 7.0, 11.8	8.5 7.8, 11.3	8.0 4.5, 12.0	9.0 4.0	8.0 6.0	2.0 0
CHAMPS Participation (hours/week)	28.9 11.6, 36.1	27.8 16.1, 34.9	21.1 13.7, 30.3	33.0 30.5	32.3 27.8	35.8 24.8
CHAMPS Walking activity (hours/week)	0.5 0, 1.5	2.1 0.5, 2.8	3.5 1.7, 5.3	1.8 0	2.3 2.3	1.8 1.0
CHAMPS Moderate intensity physical activity (hours/week)	2.8 0.4, 5.3	3.0 0, 6.2	2.0 1.3, 4.3	2.3 0	1.0 0.5	0.5 0.5
CHAMPS Vigorous intensity physical activity (hours/week)	0 0, 0.4	0.3 0, 0.5	0.3 0, 1.2	0 0	0 0	0 0

*6MWT* 6-min walk test, *10mWT* 10-m walk test (gait speed). *CHAMPS* Community Health Activities Model Program for Seniors subscales: Participation #1–40; walking items #25–28; moderate intensity physical activity = items #7, 9, 14–16, 19, 21, 23–26, 29–33, 37, 38, 40; vigorous intensity physical activity = items#14, 24, 25: hours/week = hours per week

### Identify the feasibility and acceptability of the study protocol

#### Quantitative

The recruitment rate was approximately 5 participants per month. There were no dropouts; all participants completed the follow-up assessments. All study measures were completed at each assessment time by all participants. There was limited wear time of the activity monitors and GPS devices for one participant.

Two of 18 planned sessions for the GO-OUT group were canceled due to rain. Of the 16 remaining sessions, the average attendance was 61%, with individual attendance ranging from 25 to 100%. Removing the two attendees with the lowest attendance (a couple), the average attendance of the other four participants was 80%. The GO-OUT group completed all activities for each session as per the guidelines that were prepared for the walk group leaders. GO-OUT participants started keeping track of the number of steps that they took at each session, using their own pedometers, and noted an increase in steps over the weeks. At the interactive workshop, all participants completed every station, with one participant observing one activity station, due to fatigue.

#### Qualitative

At 3 months, we conducted a focus group with three participants in the GO-OUT group (1 man, 2 women). We also scheduled interviews with three women in the control group, because a common time for a focus group could not be identified. All participants lived in the city of Winnipeg (pop. 718,000) [70]. Four themes emerged from the analysis: barriers to outdoor walking, impact of the interactive workshop, impact of the GO-OUT walking group, and feasibility and acceptance of the assessment and intervention strategies.

##### Barriers to outdoor walking

A primary internal barrier to outdoor walking described by all participants was a *lack of motivation.* Participants attributed their lack of motivation to decreased confidence in walking alone, an inability to put the knowledge of the importance of physical activity into action, and the presence of depressive symptoms.

Participants described a reluctance to join existing walking groups as shared by one person:

I’m a little hesitant because these are people who already know how to walk….And I would feel really stupid if I was [lagging] behind. (GO-OUT, participant 3)

Participants randomized to the interactive workshop-only group described a wish to have been assigned the GO-OUT program, feeling that it would have been beneficial and motivating to walk outdoors more frequently. One participant stated:

Well I guess I would put it this way for me, not having a cohort [walking group] was, or a schedule or a, decreased my ability to follow through with the walking. Like to me that is a really important piece. (Workshop, participant 3)

Participants frequently discussed fear of injury, falls, or pain and a sense that there was increased risk in walking outside, particularly in the winter (although the study occurred in summer weather). As one participant reported:

Well you can’t walk outside safely. You don’t want to fall. [while walking outside in winter] (Workshop, participant 2)

##### Perceived impact of the interactive workshop

Participants perceived a social benefit of the interactive workshop, in learning and working through stations with a group of peers. While some participants felt they learned new information at the workshop, most reported that the information provided was a reminder. They felt it was motivating and inspiring to start being more active, as shared by one individual:

And it just, the workshop was, how do you say it, gives me the inspiration to get started. It was so good. (GO-OUT, participant 2)

However, those in the interactive workshop group felt that, while the workshop increased awareness, it did not lead to meaningful behavior change. All participants were given a pedometer at the workshop, and behavior change appeared to be relative to the use of the pedometer. Pedometers were described as motivating, useful for setting goals for steps per day, and provided feedback as to how active they were. All those interviewed stated that they used their pedometers afterwards to some extent, as described by one individual:

And the pedometer, just so I had a better idea of what I was doing…And I use it all the time. (Workshop, participant 4)

The participants in the interactive workshop-only group described some increased walking and goal-setting initially, but it did not appear to be sustained. One participant suggested that for a future study, regular phone follow-up asking how much they were walking would be beneficial for the interactive workshop-only group.

##### Perceived impact of the GO-OUT walking group

GO-OUT walking group participants described the benefit of a group format in helping to address their decreased motivation to walk outdoors and to be more active. Peer support was important, and they found it motivating to see other group members improve. They stated that walking in parks in a group was positive as it felt safer than walking alone. The walking facilitators created a comfortable, non-competitive atmosphere; although walking in a group, there still had the opportunity to be challenged individually as there was more than one walking facilitator. The experiential aspect of the walking group was highlighted as important. One participant reported:

That is because then you can remember better if you actually do it. Like just somebody talking and telling you things like that, that doesn’t stick. It’s when you actually do what they are explaining and teaching you that stays with you. (GO-OUT, participant 2)

All GO-OUT group members discussed how their confidence to walk outdoors improved, leading to a sense of freedom. One participant shared:

But the main effect for me is it did exactly what I wanted….Gave me confidence to walk…It changed my way of thinking. Gave me confidence to go out there and actually walk. (GO-OUT, participant 3).

Practicing skills and increasing the walking challenges (e.g., distance, terrain) over time facilitated learning in a safe environment. GO-OUT participants identified that their walking distance and endurance improved over time and some felt that their energy improved on the day of the walk. All three described improved mood, deceased depression, and an improved sense of well-being. They linked their improved mood to an increased sense of coping and a positive outlook as described by one participant:

Getting out and doing something like walking like this, it changes your attitude. I don’t know how it all works but it does. (GO-OUT, participant 2)

GO-OUT participants stated that through participating in the study, they learned about new parks and places to walk. At the time of the focus group, all described continuing to walk outdoors and using their pedometers.

##### Feasibility and acceptance of the assessment and intervention strategies

No issues with the use of the activity monitors and GPS units for assessment were identified in the interviews. The length of time for the assessments and the type of physical assessments and self-report questionnaires were considered acceptable. Participants in the GO-OUT group enjoyed the intervention and participants in the interactive workshop group described wishing they had been allocated to the intervention walking group. There were no suggestions to improve workshop stations. Participants provided ideas for an enhanced recruitment strategy: using public service announcements and advertisements on “oldies” radio stations.

#### Identifying challenges in the pilot

Table 4 represents the changes to the protocol which were made based on the results and experience of the pilot study.

**Table 4:** Changes made to protocol based on pilot results

Challenges in pilot study	Proposed changes to study protocol
Conceptual framework	
A conceptual framework was developed based on the literature. Participants made numerous enlightening comments regarding barriers to walking outdoors and personal changes they noted during the study in qualitative interviews.	The conceptual framework was enhanced based on qualitative findings
Recruitment	
During qualitative interviews, participants suggested additional recruitment strategies.	Public service announcements and advertisements on “oldies” radio stations will be a part of the recruitment approach.
Screening	
PAR-Q+ was used as a screen for physical activity readiness	The Get Active Questionnaire will be used, as the PAR-Q+ has since been replaced by the Get Active Questionnaire
Randomization	
A couple wanted to be in the same group.	Because participation as a couple may be a facilitator of outdoor walking activity (primary outcome), participants will be stratified as singlets or dyads prior to randomization
Had planned to stratify participants by gait speed (< 0.8 m/s vs ≥ 0.8 m/s); however, no participants walked slower than 0.8 m/s.	Will not stratify by gait speed.
Data collection, outcomes and analysis	
One participant did not meet minimum wear time for activity monitor and GPS.	Participants will be reminded verbally when receiving the activity monitor and in writing on an activity log that they need to wear the monitor morning to night and for a minimum of 10 h per day. IF they do not achieve a min 4 days of wear time, they will be asked to wear the monitor on additional days.
Participants had very low activity by activity monitors.	Analysis of activity monitor and GPS data will define bouts as “Lifestyle 5 min” and will also present daily sporadic minutes of Lifestyle MVPA.
	Analysis of cadence will also be used to better describe continuous purposeful walking
Time intensive to match GPS and activity bouts. Some bouts difficult to determine if indoors or outdoors.	Introduce an outdoor walking time log, to assist with identification and analysis of outdoor activity.
Did not objectively quantify extent and intensity of walking activity during the outdoor walking sessions (process indicator).	GO-OUT participants will be asked to wear the activity monitor/GPS during two outdoor walking group sessions to evaluate extent and intensity of walking activity and compare across sites.
Scores on the Berg Balance Scale and Geriatric Depression Scale were similar across study evaluations.	The Berg Balance Scale and Geriatric Depression Scale will not be used. The Berg Balance Scale will be replaced with the Mini-BESTest [73], which may be more sensitive to change resulting from the GO-OUT intervention. The Emotional Well Being subscale of the RAND-36 will substitute for the GDS as a measure of emotional health.
Intervention	
One participant suggested that for a future study, regular phone follow-up asking how much they were walking would be beneficial for the workshop group.	The workshop group will have weekly phone reminders to re-iterate information learned in the workshop and to encourage outdoor walking.
Duration of GO-OUT intervention was 9 weeks due to the start time of project.	GO-OUT program will be 12 weeks.
For personal safety, GO-OUT participants did not like one park with isolated parking and forested walking trails with few other walkers.	Ensure that parks used for GO-OUT do not have isolated parking areas and have numerous walkers in the area.

## Discussion

Participants were below norms for age in many outcomes that are considered to be internal barriers to outdoor walking. The mean number of steps/day taken by participants in our study was 3893, which corresponds with the average steps/day measured in a large sample of Americans aged 66–75 [71]. The 6MWT results, however, were below norms for the majority of participants [33]; this suggests that while individuals in our study accumulated volumes of activity (steps/day) that were comparable or greater than age and sex-matched peers, they likely accumulated these steps in short bouts of activity and did not have exercise tolerance levels that would allow them to excel in the 6MWT. Comfortable gait speed at baseline was slightly below norms for age and sex [33]. Median baseline values for all RAND-36 subscales were lower than Canadian norms for age for all participants, except for emotional well-being in the interactive workshop group [72]. Median baseline values of the 30-s sit-to-stand test were lower than mean scores in older adults with low activity levels [36].

### Identify the optimal analysis method of outdoor walking activity

The low 6MWT scores correspond with the participants being a low-active group. For this reason, we analyzed the data using a 5-min Lifestyle bout definition, and even using this relatively low threshold, participants recorded few walks that lasted 5 min or more (Table 2). It is evident that people did walk indoors, and had a small amount of outdoor walking. There are some ‘unknown location’ bouts of walking, which demonstrate some of the challenges in analyzing outdoor walking with GPS. With the exception of one person, all participants had enough days of wear time to enable analysis, suggesting that asking people to wear the activity monitor and GPS for 8 days is suitable. Progress was made in identifying a feasible method of analyzing outdoor walking. We are confident that the activity monitor and GPS analyses that we utilized were appropriate. Overall, it was very low-active group; the Lifestyle 5 min bouts represent purposeful walking for this less active group. In future analyses, we will also use cadence as part of the analysis. Cadence can be used to define continuous purposeful walking, e.g., 5 min bouts at a minimum cadence level.

### Evaluate the preliminary effect and safety

The total self-perceived time walked per week, according to the CHAMPS, appeared to improve over time in the GO-OUT group; walking time increased at 3 months in the interactive workshop group, but was not sustained. Walking self-efficacy (ASCQ) improved in the GO-OUT group. The interactive workshop group had higher 6MWT, ASCQ, 10mWT, and physical function at baseline than the GO-OUT group. All participants had Berg balance scores at the higher (better balance) end of the scale. Keeping in mind the small sample size, there were a few outcomes at 3 months that appeared to improve in the GO-OUT group, and decrease in the workshop group: endurance (6MWT), role limitation due to emotional problems (RAND-36), physical function (RAND-36), and emotional well-being (RAND-36). Findings are also similar to a previous study that demonstrated improved HRQL and self-reported walking activity after a peer-led neighborhood walking group [14]. External barriers of community walkability were assessed at baseline with the NEWS. Subscales reflected on average, moderate neighborhood walkability.

In comparing objective physical activity data to self-reported walking and moderate/vigorous activity time in the CHAMPS, there were some similarities. For example, activity monitors worn by the GO-OUT group at 6 months recorded approximately 30 min/day of sporadic Lifestyle MVPA which (when multiplied by 7 days) is very similar to the median 3.5 h/week reported spent in walking activities. However, these similarities between objective and self-reported physical activity levels did not apply to the control group. The timing of the two assessments was slightly different: the CHAMPS refers to activity in a typical week over the past 4 weeks, whereas the monitor was worn in the week immediately after answering the CHAMPS questionnaire.

The qualitative interviews supported the increased self-reported walking in the GO-OUT group, as well as improved endurance (6MWT), HRQL (RAND-36), and improved ambulatory self-confidence.

The GO-OUT intervention appeared to be safe. The interventions were supervised, and falls that occurred outside of intervention time did not appear to be related to the interventions, but rather to activities the participants had previously planned. However, we need to evaluate this further with a larger study. There could be a possibility that as people begin to feel more confident, they take chances they would not have previously taken.

### Identify the feasibility and acceptability of the study protocol

The protocol was feasible to implement as noted by the ability to collect all outcomes at multiple time points and successful completion of the interactive workshop and the GO-OUT intervention by all participants; this was also supported by the qualitative data. Participants did not identify problems with using the GPS and activity monitors, they found the questionnaires reasonable, and those in the GO-OUT group found it beneficial and enjoyable.

#### Limitations

The small sample size of nine is a limitation to this pilot study. There was only one person that used a walking aid, making it difficult to know how others with walking aids would participate in the GO-OUT intervention. Since it was not possible to blind the participants, there is the potential risk of performance bias. The inclusion and exclusion criteria were quite strict to ensure safety of the participants in the pilot. This may limit generalizability of the pilot results.

## Conclusions

The objectives of the study were met. An analysis method combining accelerometry and GPS to determine outdoor walking was developed for an older adult population who infrequently walked outdoors. The GO-OUT intervention and interactive workshop were safe, and changes in physical capacity and HRQL appear to have occurred, given the small number of participants. Participants accepted the frequency and content of the evaluation sessions. Participants shared their perceptions of barriers to outdoor walking, impact of the interactive workshop and GO-OUT walking group, and feasibility and acceptance of the assessment and intervention strategies. A full-scale four-site study is underway.

## Abbreviations

6MWT: Six-minute walk test
ASCQ: Ambulatory Self-Confidence Questionnaire
CHAMPS: Community Health Activities Model Program for Seniors
GO-OUT: Getting Older adults OUTdoors
GPS: Global positioning system
HRQL: Health-related quality of life
MVPA: Moderate-to-vigorous physical activity
NEWS: Neighbourhood Environment Walkability Scale
